# A Lightweight Detection Method for Remote Sensing Images and Its Energy-Efficient Accelerator on Edge Devices

**DOI:** 10.3390/s23146497

**Published:** 2023-07-18

**Authors:** Ruiheng Yang, Zhikun Chen, Bin’an Wang, Yunfei Guo, Lingtong Hu

**Affiliations:** School of Automation (School of Artificial Intelligence), Hangzhou Dianzi University, Hangzhou 310018, China; 212060164@hdu.edu.cn (R.Y.); 211060043@hdu.edu.cn (B.W.); gyf@hdu.edu.cn (Y.G.); 222060235@hdu.edu.cn (L.H.)

**Keywords:** remote sensing, lightweight neural network, CNN accelerator, field-programmable gate arrays (FPGAs)

## Abstract

Convolutional neural networks (CNNs) have been extensively employed in remote sensing image detection and have exhibited impressive performance over the past few years. However, the abovementioned networks are generally limited by their complex structures, which make them difficult to deploy with power-sensitive and resource-constrained remote sensing edge devices. To tackle this problem, this study proposes a lightweight remote sensing detection network suitable for edge devices and an energy-efficient CNN accelerator based on field-programmable gate arrays (FPGAs). First, a series of network weight reduction and optimization methods are proposed to reduce the size of the network and the difficulty of hardware deployment. Second, a high-energy-efficiency CNN accelerator is developed. The accelerator employs a reconfigurable and efficient convolutional processing engine to perform CNN computations, and hardware optimization was performed for the proposed network structure. The experimental results obtained with the Xilinx ZYNQ Z7020 show that the network achieved higher accuracy with a smaller size, and the CNN accelerator for the proposed network exhibited a throughput of 29.53 GOPS and power consumption of only 2.98 W while consuming only 113 DSPs. In comparison with relevant work, DSP efficiency at an identical level of energy consumption was increased by 1.1–2.5 times, confirming the superiority of the proposed solution and its potential for deployment with remote sensing edge devices.

## 1. Introduction

The use of object detection technology with remote sensing images is aimed at obtaining a specified region of interest from an image based on a specific algorithm to determine its location and category. The early target detection algorithms for remote sensing images employed artificial design features, but they could not capture abstract semantic features effectively. Thanks to the development of deep learning and computer vision over the past few years, numerous high-precision object detection models based on convolutional neural networks (CNNs) have been proposed for remote sensing image processing, which has notably improved the efficiency of feature extraction from remote sensing images, and object detection from remote sensing images based on deep learning has also developed rapidly. It has been extensively employed in ship detection [1,2,3], meteorological environment detection [4,5], husbandry monitoring [6,7], road extraction [8,9,10], and other fields.

Conventional image processing algorithms should transmit remote sensing images back to the ground station server for centralized processing [11]. This processing method is significantly limited by the computing power of the ground station server and the communication delay between the remote sensing platform and the ground station and cannot keep up with the speed of remote sensing image acquisition, resulting in considerable amounts of image data being idle or even discarded and a serious waste of resources [12]. Edge computing can be adopted to address the inherent delay problem in centralized processing methods: the processing of remote sensing image data can be pushed from the ground station server to the remote sensing edge device, and image processing algorithms can be applied close to the data source to avoid the computing power bottlenecking arising from transmission delay. Edge computing has the advantages of low latency, high feasibility, and good real-time performance. Accordingly, remote sensing image processing algorithms with high-computational consumption characteristics are more suitable for migration to edge devices. Detection algorithms for remote sensing edge devices have been actively researched recently [13,14,15,16].

As CNNs have been continuously advancing, the increasing number of network layers has enhanced the network feature extraction ability, and the number of parameters and the size of the model have been also increased. Many researchers have investigated how to reduce the parameters and calculations of CNN algorithms. One method is to design a lightweight structure for the network (e.g., ShuffleNet [17] and MobileNet [18]), and various researches have also made improvements based on these lightweight networks [19,20]. Zhao et al. [19] replaced the backbone of YOLOv4 with ShuffleNetv2. Saiful Bahri et al. [20] replaced the backbone of the SSD network with MobileNetv2. Both achieved high accuracy with a smaller size. However, the above-mentioned networks require additional hardware optimization if they are to be deployed in edge devices because the depth-wise separable convolution they adopt causes a large number of memory access events in the edge device hardware, resulting in problems such as high energy consumption and low energy efficiency. Another effective method refers to pruning the models [21,22]. Gale et al. [21] proposed a sparse optimization strategy with ImageNet and well reduced the number of parameters. Jorge et al. [22] removed nearly 99.5% of parameters while keeping the networks trainable. However, the above-described methods significantly affect the accuracy of CNN models, and they may not even conform to the needs of practical applications involving edge devices.

Thus, how networks can be lightweight and deployed with edge hardware platforms while ensuring high precision has become a new challenge [23]. To tackle this problem, some researchers have designed lightweight network architectures suitable for edge devices [24,25]. Ma et al. [24] proposed a lightweight detector for remote sensing images. Fu et al. [25] proposed a fast detection model for UAV edge devices and achieved high accuracy. However, these lightweight methods often only meet the availability requirements of edge device processing but cannot guarantee real-time detection performance. Accordingly, the acceleration of network inference is necessary. 

Common high-performance acceleration platforms include graphics processing units (GPUs), central processing units (CPUs), and so forth. However, these devices are difficult to deploy in embedded edge devices due to their high power consumption, and thus field-programmable gate arrays (FPGAs), and application-specific integrated circuits (ASICs) with low power consumption have been widely employed to enhance computing performance. The ASIC is characterized by a high degree of customization, high R&D costs, and a long R&D cycle, and thus it cannot be applied to object detection, where the model is constantly changing [26]. FPGAs include considerable programmable logic units while exhibiting a static global reconfiguration, the ability for pipeline parallel computing, and high energy efficiency, allowing them to be applied to the object detection field where the algorithm model is changing rapidly and must conform to the energy efficiency requirements of edge devices [27].

However, existing FPGA accelerators tend to trade area for performance, i.e., exploiting more hardware resources to obtain higher computing throughput, whereas energy efficiency is rarely increased. For instance, Zhang et al. [28] proposed a high-performance accelerator for deep convolution neural networks, consuming 2833 DSPs and achieving a power consumption of 26 W. Li et al. [29] proposed an efficient remote sensing images detection framework and its high-throughput accelerator based on the FPGA, and a power of 19.52 W and 1152 DSPs were consumed. Such hardware resource consumption is unacceptable on remote sensing edge devices with strict power limits. Thus, an energy-efficient CNN accelerator with less hardware resource consumption should be urgently developed. In addition, existing research on FPGA accelerators has only deployed the target detection network at the hardware level, whereas the necessity of lightweight algorithms has been ignored [30,31]. Sledevič et al. [30] proposed a CNN core architecture for VGG networks. Hareth et al. [31] proposed a 2D PE array to accelerate convolutional operations in AlexNet. To conform to the deployment requirements of these networks, researchers have to use more hardware resources, which also means higher power consumption. Therefore, if the accelerator can be co-optimized at the software and hardware levels, a higher throughput may be achieved with lower power consumption.

Following the above discussion, a lightweight remote sensing image detection model and its high-efficiency hardware acceleration solution are proposed. The overall contributions are summarized as follows:

- Based on YOLOv3, several network lightweight methods are proposed:
- Using lightweight GhostNet as a feature extraction network.
- Compressing the model by channel pruning and restoring the precision by knowledge distillation.
- Performing fixed-point quantization of parameters to reduce the difficulty of network hardware deployment.
- An energy-efficient CNN accelerator is designed based on the lightweight methods:
- According to the characteristics of the optimized network, different convolutional computation engines for different modules are designed to increase the efficiency of computing resources.
- The SE attention mechanism in the network in hardware friendly aspects is optimized to expedite the network inference.
- For data storage optimization, a dual cache mechanism and convenient memory access storage method are adopted to reduce memory access overhead.

The rest of this study is organized as follows. In Section 2, the specific principles and methods of the solution are introduced, including network lightweight and CNN accelerator design. In Section 3, the experimental results and performance evaluation are presented. In Section 4, the innovation and limitations of this solution, as well as its possible applications and implications are discussed. In Section 5, a conclusion of this study is drawn.

## 2. Materials and Methods

### 2.1. Improved Lightweight Ghost-YOLOS Network

#### 2.1.1. Network Structure

The YOLO (You Only Look Once) algorithm solves object detection as a regression problem [32]. This algorithm conforms to the main principle of outputting the class probability, location, bounding box, and other information of the respective object in the input images using an independent end-to-end network. The YOLOv3 inherits the advantages of a fast detection speed, high-detection accuracy and strong generalization ability of the previous two generations of the YOLO network, while adding three object detection scales that are capable of detecting large-scale objects, medium-scale objects and small-scale objects [33]. Nevertheless, the parameters amount of YOLOv3 is excessively large, and the model occupies excessive resources, such that it is difficult to transplant to edge devices for real-time data processing. The original YOLOv3 model structure is improved to reduce the complexity of the model and parameters.

**Figure 1:**
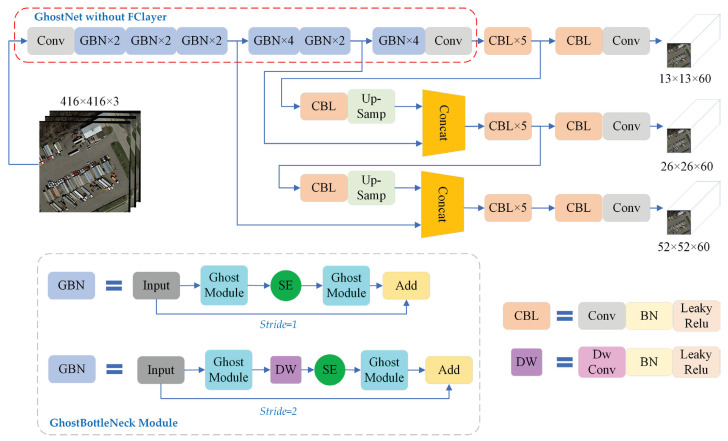
Improved Ghost-YOLO network structure. SE module means Squeeze and Excitation module, which is only present in the GBN structures at layers 4, 5, 10, 11, 12, 14 and 16 in GhostBottleNeck. Dw Conv module means depthwise separable convolution.

The original YOLOv3 network comprises two parts, i.e., backbone and YOLO head. In this study, the GhostNet network structure is employed, and the Darknet-53 backbone feature extraction network in YOLOv3 is replaced with it. Figure 1 presents the optimized network structure.

**Figure 2:**
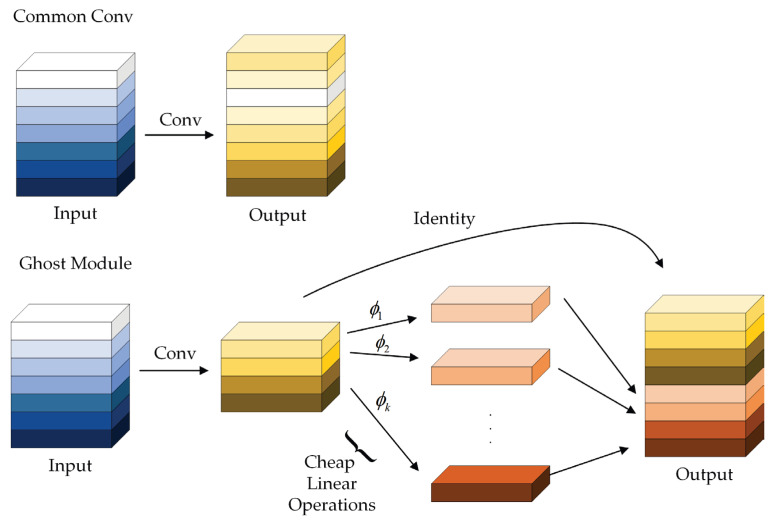
Comparison of common convolution and ghost module.

GhostNet network structure is proposed by Han et al. [34]. The main improvement conforms to Ghost module, which can make the whole network of the amount of computation and the number of parameters notably reduced without changing the convolutional output feature map size and channel size. The comparison between conventional convolution and Ghost module is shown in Figure 2. 

If the input feature diagram is expressed as H×W×C, then the output is $H^{\prime }\times W^{\prime }\times M$; the input is divided into n layers, the size of the convolution kernel is k×k, then the calculation amount of common convolution and Ghost convolution is shown in Equations (1) and (2). From the calculation of the Ghost module, it can be found that, through the module, replacing the common convolution can effectively reduce the network computational complexity. Moreover, the GhostNet network also introduces an SE attention mechanism module into its GhostBottleNeck module structure, which tends to increase the specificity and utilization of features.

(1) $$CALC_{common\_conv}=H^{\prime }\times W^{\prime }\times M\times k\times k\times C$$

(2) $$CALC_{ghost\_conv}=H^{\prime }\times W^{\prime }\times \frac{M}{n}\times k\times k\times C+(n-1)\times H^{\prime }\times W^{\prime }\times \frac{M}{n}\times k\times k$$

Although the number of parameters of the YOLOv3 g network structure is significantly less than that of the original network, it is still difficult to deploy in low-cost embedded devices. Accordingly, the overall network structure should be compressed to reduce the resources and memory occupied by it.

#### 2.1.2. Model Pruning

**Figure 3:**
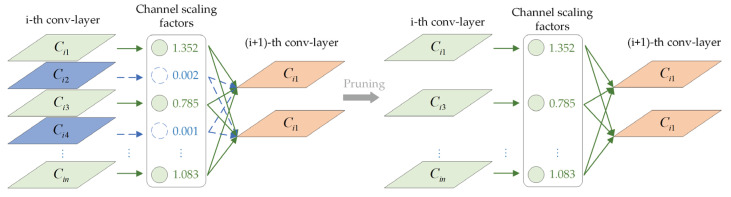
Channel pruning based on BN layer. The channel with scaling factor 0 or close to 0 is deleted and the connection is removed.

To compress the size of the model, this study first prunes the Ghost-YOLO network. The channel pruning method, based on the BN layer, is adopted for network model pruning [35]. Figure 3 presents the pruning method. The network model is modified by constantly adjusting the compression ratio of channel pruning. Moreover, the spatial complexity of the cropped model and the requirements of edge device hardware deployment are reduced.

#### 2.1.3. Knowledge Distillation

**Figure 4:**
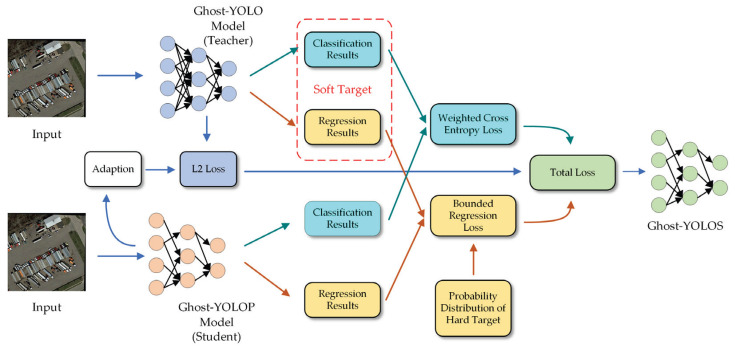
Improved knowledge distillation with regression results.

Channel pruning will cause some loss to the accuracy of model inference prediction. Thus, the knowledge distillation method is employed in the precision restoration and fine-tuning of the trimmed Ghost-YOLOP network. The pruned model, Ghost-YOLOP, serves as the student model, and the unpruned model, Ghost-YOLO, is adopted as the teacher model. For the classification loss of Ghost-YOLO, the knowledge distillation method employed by Hinton [36] for classification is adopted. For the loss of bounded regression, Chen’s [37] regression loss method for Faster RCNN is used. The process is presented in Figure 4. Compared with the conventional knowledge distillation method [36], the calculation of the loss function of the improved knowledge distillation method should calculate the classification loss, the regression loss, as well as the intermediate layer loss.

#### 2.1.4. Network Quantization

The model obtained by channel pruning and knowledge distillation employs 32-bit floating-point data. Compared with complex floating-point arithmetic, fixed-point arithmetic in the FPGA and other devices, this new model exhibits the advantages of less hardware resource consumption (e.g., DSP and LUT), a higher working frequency and fast arithmetic speed, such that the floating-point data of the model should be converted into fixed-point data. Moreover, the research shows that [38,39] a 16-bit fixed-point quantization for the convolutional neural network model is capable of accelerating the model while maintaining good recognition accuracy. Thus, in this study, dynamic fixed-point 16-bit quantization [40] is adopted to quantify the weight and bias parameters of the model, input and output feature graphs and intermediate results, with the aim of reducing the bit width of data and the amount of computation and data transmission. The above-described method primarily seeks the optimal exponent-marker through traversal, as expressed in Equation (3).

(3) $$\exp_{Q}=\arg\min\sum_{i=0}^{n}\left|Q_{float}^{i}-Q_{\left(bw,\exp_{w}\right)}^{i}\right|$$

where $\exp_{Q}$ denotes the exponent-marker with the least loss of precision after quantization, n represents the amount of data that should be quantified, $Q_{float}^{i}$ expresses the original floating-point value of the i-th number, $Q_{\left(bw,\exp_{w}\right)}^{i}$ denotes the i-th number under bit width bw and exponent-marker $\exp_{w}$, which is quantized to a fixed-point number first, and then converted back to a new floating-point value.

### 2.2. FPGA-Based Hardware Accelerator

**Figure 5:**
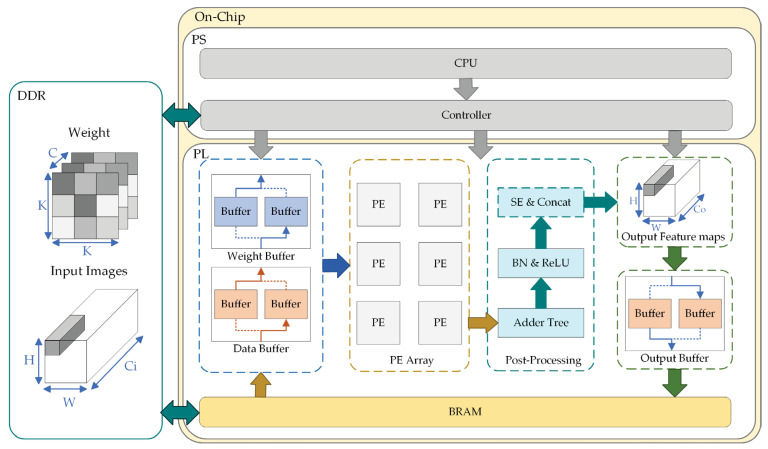
CNN accelerator architecture. The main part comprises buffer, processing engines, post-processing module and output module. The buffer section adopts dual cache design. The accelerator operates in a modular sequence in most cases. The symbols “H, W” mean the height and weight, the symbols “Ci, Co” mean the number of input and output channels, and the number of channels, and the symbol “K” means the size of convolution kernel.

In this section, the proposed FPGA-based hardware accelerator is elucidated, whose block scheme is shown in Figure 5. The system comprises three parts, i.e., Double Data Rate (DDR) Synchronous Dynamic Random Access Memory (SDRAM), Processing System (PS) and Programmable Logic (PL).

Despite the lightweight processing of neural networks, the memory footprint of the network (approximately 16 Mbit) is still much larger than that of low-cost FPGA’s available on-chip memory (less than 1 Mbit). Accordingly, we use DDR to store the feature maps and weights in the network inference process. 

The PS part controls the whole accelerator of the PL part by transmitting instructions through the data interface module, such as controlling the enable of each module to execute the streaming operation in the network model. Moreover, BRAM receives the optimal weight data after training on the PC side and the original input image in batches through the data interface, and writes the weight and image data into the data transmission buffer. To realize the efficient utilization of bandwidth, data, weight, and output buffers, it adopts a “ping-pong” structure to transmit data without any slack. 

The PL part is used for convolutional network calculation. It is mainly composed of some Processing Elements (PE), which can carry out convolution calculation of different modes with the received data. The other part is post-processing. First, the adder tree is used to sum and merge the data after the convolution operation. Then, the obtained results are processed by batch normalization (BN) and activation, which uses Leaky ReLU function to determine the threshold of pixel value. Lastly, according to the current convolution mode, is the Ghost Module, which is used to determine whether to concatenate the identity of the input feature map or use the SE module.

**Figure 6:**
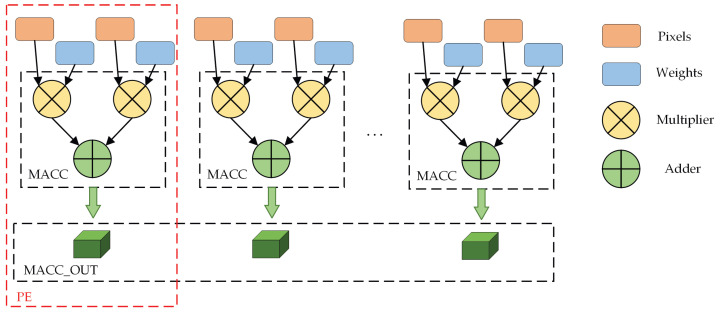
CNN accelerator PE array design. Pixels and weights are from the previous buffers.

The PE array primarily comprises some Multiplication Accumulators (MACC), and its architecture is illustrated in Figure 6, where the respective PE unit processes the multiplication of an input feature map and a set of weights.

**Figure 7:**
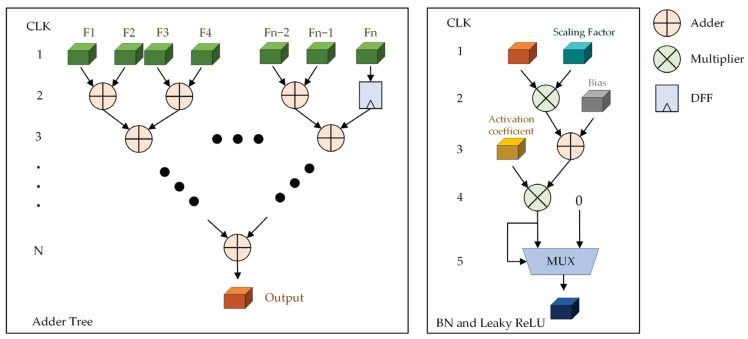
CNN accelerator post-processing module design. The symbol “Fx” indicates the x-th output feature, which comes from the output of the PE array. Scaling factor and bias are used for batch normalization. Activation coefficient is the coefficient of activation function. The symbol “…” indicates that we omit the repetitive process.

Figure 7 shows the hardware implementation details of the post-processing module. The adder tree is mainly composed of adders and flip-flops, and its main function is to accumulate the processing results of the PE unit. If the parallelism of PE array is n, then the clock cycle required for processing is ${N=\log}_{2}n$. The BN and leaky ReLU module is mainly composed of multipliers, adders, and multiplexers. The scaling multiple, bias and activation coefficient in the module are stored in the BRAM in advance and read in turn during calculation.

#### 2.2.1. Data Storage Optimization

In the Ghost-YOLOS network, the computing of the respective layer requires frequent reading and writing of data to the off-chip DDR, which will lead to the degradation of hardware accelerator computing performance due to data transmission delay. Thus, the data reading and storage methods should be optimized.

**Figure 8:**
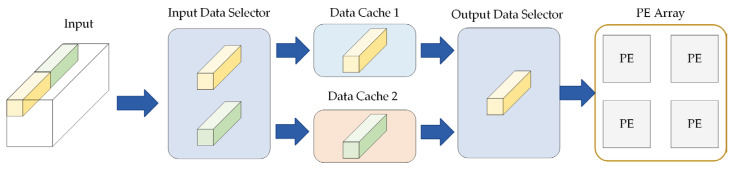
Dual cache design.

Dual cache is developed to reduce the transfer time for reading data from DDR to on-chip and for writing computed results from on-chip back to DDR. The specific structure of dual cache design is presented in Figure 8. On the first and second clock cycle, the input data is stored in cache 1 and cache 2 through the input data selector. Moreover, the data in cache 1 is output to the convolution arithmetic unit through the output data selector. In the third clock cycle, the input data is stored in cache 1, and the previous operations are repeated successively to achieve data pipeline-processing and shorten the time of data transmission from off-chip to on-chip. The size of input cache and output cache is calculated by Equations (4) and (5).

(4) $$N_{in}=\frac{T_{ix}\times T_{iy}\times d_{w}}{M_{BRAM}}\times T_{n}$$

(5) $$N_{out}=\frac{T_{ox}\times T_{oy}\times d_{w}}{M_{BRAM}}\times T_{m}$$

where $M_{BRAM}$ denotes the size of data that can be stored in a piece of BRAM, and $d_{w}$ represents the data bit width in I/O feature maps.

The total delay time for completing a convolution operation with or without the dual cache design is shown in Equations (6) and (7).

(6) $$T_{without\_dc}=T_{read}+T_{compute}+T_{write}$$

(7) $$T_{with\_dc}=\max(T_{read},T_{compute},T_{write})$$

The Ghost-YOLOS network has considerable convolution operations and frequent DDR memory access. By consuming twice the BRAM logic resources, the dual cache design can significantly reduce latency due to data transfer, thereby improving accelerator performance.

The method of array segmentation is adopted to store the data of feature maps and weights in the BRAM to solve the problem that excessive data may lead to bottlenecking the data access. The above-described method is capable of increasing the local memory bandwidth, and it can be combined with the dual cache design to enhance the performance of the system’s ability to read and write data.

**Figure 9:**
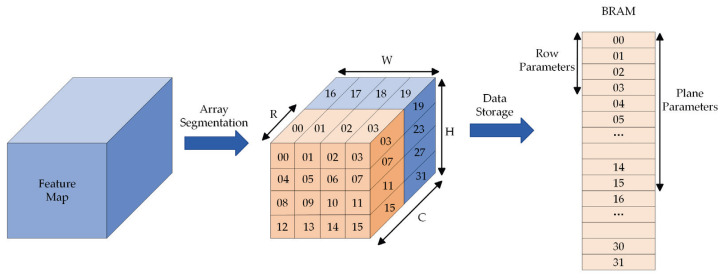
Block storage of input feature map. The symbols “H, W, C” mean the height, weight and the number of channels, and the symbol ”R” means the number of channels stored in BRAM.

Figure 9 presents the block storage of input feature map data. The input feature map falls into blocks on the width, height, and the number of channels. The respective block of data is stored in BRAM in accordance with the order of reading. In the BRAM, the data is stored from low address to high address. Furthermore, the row parameter index and the plane parameter index of the input feature map data are stored in the register.

**Figure 10:**
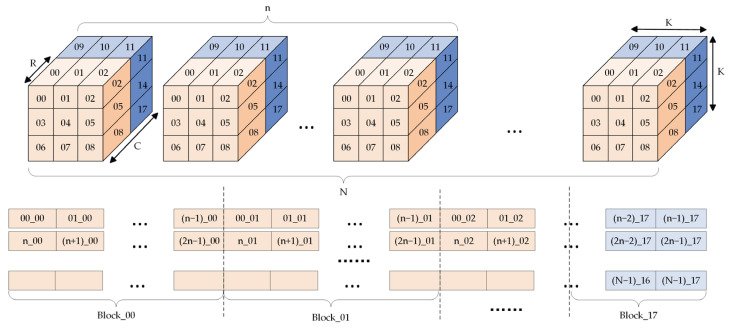
Block storage of weights. The symbols “C, K” mean the number of channels and the size of convolution kernel, and the symbol ”R” means the number of channels stored in BRAM.

Corresponding to the storage format of the input feature map, the respective weight in the convolutional kernel is divided and stored in the identical form to match the data of the input feature map for the convolution operation. The weight is iterated and follows the order of the number of input channels, the number of output channels, filter height and filter width, and is stored in the block cache (Figure 10).

#### 2.2.2. Convolution Architecture with High Parallelism

Convolution calculation occupies most of the calculation in the convolutional neural network, so PE array is the core component of the FPGA convolutional neural network accelerator, and how to use the resources of the PE array is an important indicator of the performance of the hardware accelerator. In the Ghost-YOLOS network, there is not only common convolution, but also depth-wise separable convolution, which is in the GhostBottleNeck module. Accordingly, to make full use of hardware resources and complete computation with a high-parallelism degree, this study designs different ways of using the PE array’s resources for different convolution modes.

**Figure 11:**
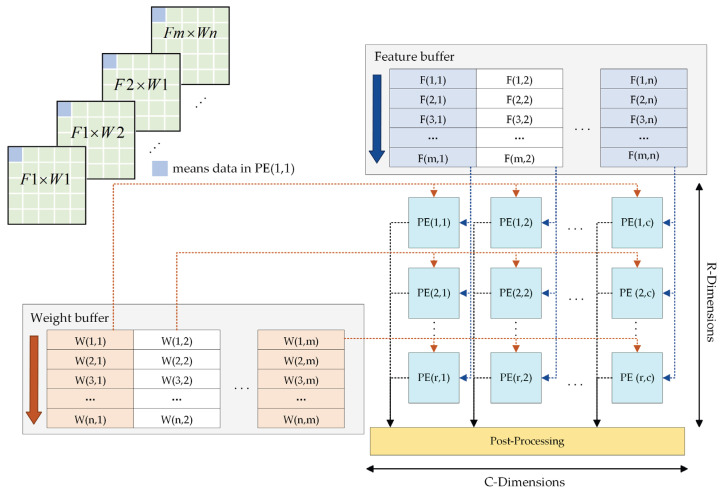
PE array architecture in common convolution mode.

For common convolution, the PE array employs parallel computation at the channel level, and the architecture is illustrated in Figure 11. In the identical clock cycle, the respective row of the PE array reads the convolve window at the identical position on the input feature map of R channels, whereas the respective column reads the convolution kernel weights of C input feature maps and then performs the convolution calculation in pairs. The computing resources employ a total of R×C groups of the PE units. After K×K clock cycles, the convolution results of a set of convolve windows and convolution kernels are determined, and then the convolve window is moved for all convolution calculations on the current feature map before inputting the next channel feature map. 

For depth-wise separable convolution, the design for common convolution cannot exhibit a high degree of parallelism. Depth-wise separable convolution comprises depth-wise convolution and pointwise convolution [41]. For depth-wise convolution, the respective output feature map is generated by convolving the single-channel input feature map with the corresponding unique convolution kernel. Thus, compared with the conventional common convolution, the channel parallel features, scalable by depth-wise convolution, are reduced by one dimension. 

**Figure 12:**
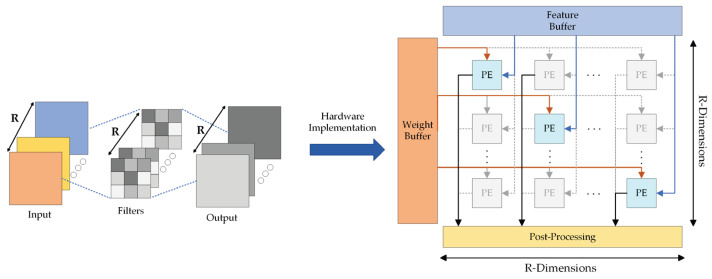
Depth-wise convolution and its application in common convolution mode. The symbol “R” means the number of channels.

Figure 12 presents the process of depth-wise convolution and its application in the common convolution PE array. In the PE array, only the input channel feature maps and weights of the PE units on the diagonal are corresponding. As indicated by the above result, only R computing units are adopted in the depth-wise convolution calculation process, and R×(R−1) computing units exhibit the non-enabled state, such that the maximum hardware utilization efficiency is not achieved.

**Figure 13:**
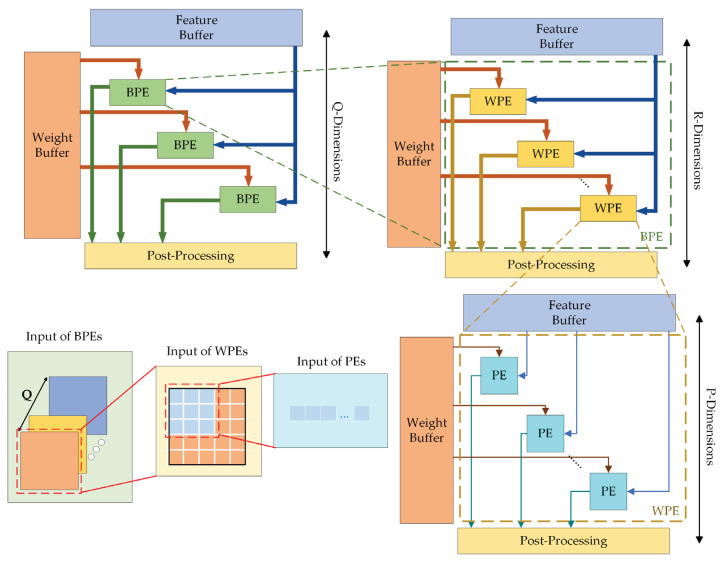
PE array architecture in depth-wise convolution mode. The symbol “Q” means the parallel factor of different input images.

Accordingly, to ensure that the computing engine shares the same PE array architecture with the common convolution in depth-wise convolution, and to ensure higher efficiency of computing resources during operation, the deep convolutional mode carries out additional data management through the controller, as shown in Figure 13. 

First, the PE array is divided into Block Processing Elements (BPE) with a parallelism degree of Q, where Q is the parallel factor of different input images. Then, each BPE obtains the feature maps of different input channels and the weights of data and corresponding channels from the feature map and weight buffer, respectively. Meanwhile, BPE contains R group of Window Processing Element (WPE) for multiplication calculation. Computations of a single sliding window are processed in parallel in each WPE. 

In the identical clock cycle, the computational architecture first loads R×P weights from the weight buffer per column. When the channels of the input feature map remain unchanged, the identical batch of weights are employed for the respective convolution calculation, such that the weights can remain unchanged. Next, P pixels of a single convolve window on R input channel feature maps of Q allows different images to be read from the data buffer, respectively, and transferred to different PE’s for multiplication calculation. Thus, the parallel multiplication calculation of the PE array can be increased to P×R×Q. The moving mode of the convolve window also gives priority to traversing the whole feature map, and then calculating the feature map of the next set of input channels until Q different input images have completed the convolution operation.

#### 2.2.3. SE Module Hardware Optimization

**Figure 14:**
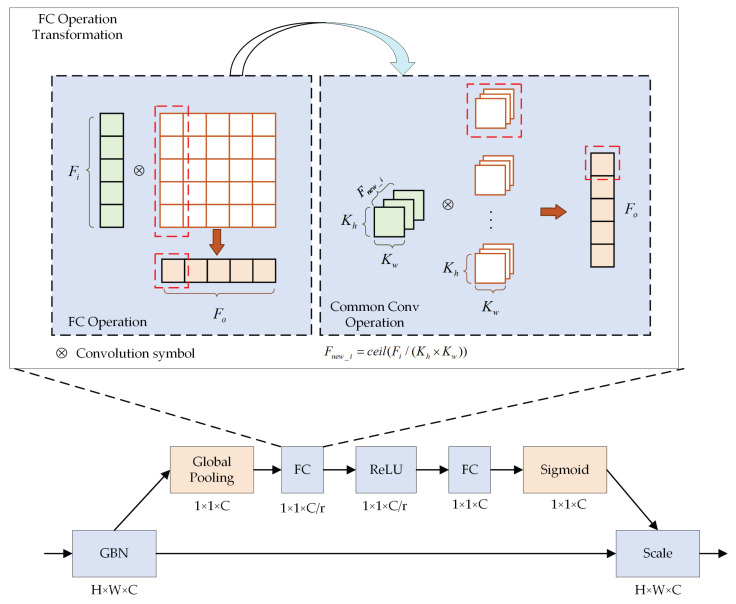
SE module in the Ghost-YOLOS network and FC operation optimization. $F_{i}$ and $F_{o}$ means input and output feature map. $F_{new\_i}$ means the feature map after transformation. $K_{h}$ and $K_{w}$ mean the width and height of the converted convolution kernel.

The SE attention mechanism is critical in GhostBottleNeck, and it appears in layer 4, 5, 10, 11, 12, 14, 16 GBN modules of the Ghost-YOLOS network. The basic architecture of the SE attention mechanism is illustrated in Figure 14, wherein both FC layers are equivalent to an unbiased linear transformation of the input of the previous layer, which can directly use the convolution computing structure mentioned in Section 2.2.2 for parallel operation, and the ReLU function can be implemented by a selector in hardware. Accordingly, it is the global pooling layer and the Sigmoid function that require hardware optimization.

**Figure 15:**
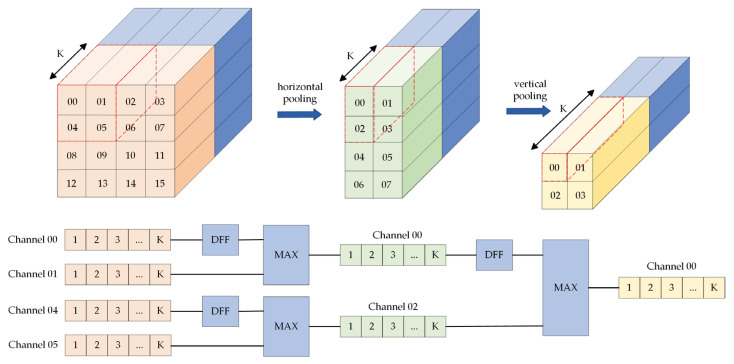
2D pooling separation of max pooling. DFF is used to cache data. MAX is used to calculate the maximum value. The symbol “K” means the number of channels for pooling operation.

Global pooling refers to a squeeze operator in the SE module. Two methods (i.e., average pooling and max pooling) are available for the global pooling module, with little difference in the effect on the result [42], whereas the operation of the two methods turns out to be highly different in hardware. In general, average pooling primarily employs multiplication and addition operations, which are difficult to complete in a clock cycle, whereas max pooling only comprises one logical operation, i.e., comparing the size of input data, which has simple logic and is suitable for running in the FPGA. Thus, we choose to use max pooling and the two-dimensional (2D) pooling separation method to optimize its operational logic, and its hardware implementation architecture is shown in the Figure 15.

Compared with the conventional 2D pooling operation implemented in hardware, one-dimensional (1D) operation has more concise control logic, can avoid a lot of redundant calculation, and is more conducive to hardware implementation. Accordingly, we decompose the 2D pooling into 1D horizontal pooling and 1D vertical pooling. In this module, a flip-flop is set to cache the data after horizontal pooling. Moreover, pooling operators of multiple windows can be operated simultaneously to speed up the global pooling process.

To further accelerate, in addition to the parallel pipeline of multiple pooling windows, the parallel operation of dataflow constraint horizontal pooling and vertical pooling is also adopted. The parallel pipeline based on the dataflow constraint does not need to wait for horizontal pooling to complete before vertical pooling. If the output of the horizontal pooling can be passed to the vertical pooling for operation, both work simultaneously, notably improving the efficiency of the operation.

**Figure 16:**
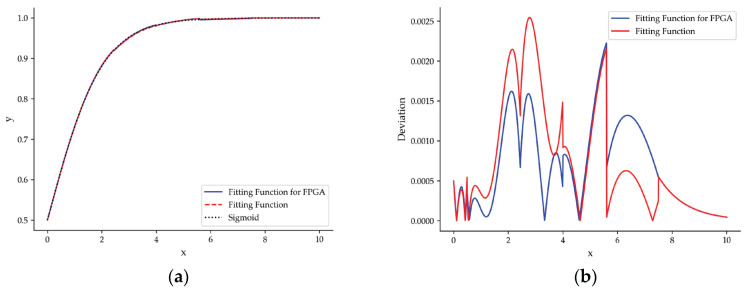
(**a**) Comparison between Sigmoid function and fitting function. (**b**) The deviation of the fitting function and the Sigmoid function.

Sigmoid function is one of the activation functions commonly used in neural networks, but its exponential property makes it difficult to implement in hardware. Thus, we perform a piecewise quadratic approximation to increase its hardware friendliness, where the coefficients can be stored in the on-chip BRAM in advance. We divided the positive semi-axis of Sigmoid function into six subintervals, and the function representation after piecewise quadratic approximation is shown in Table 1. In the FPGA, the division operation can be implemented using shifters to reduce resource consumption. The maximum deviation from the original function after approximation falls into a scope of 0.003, as shown in Figure 16.

## 3. Results

### 3.1. Experimental Settings

#### 3.1.1. Dataset

To evaluate the remote sensing performance of the Ghost-YOLOS lightweight model and verify the feasibility of the CNN hardware accelerator results, aerial DOTAv1.0 and UAV Visdrone2019 datasets were used for the experimental evaluation.

**Figure 17:**
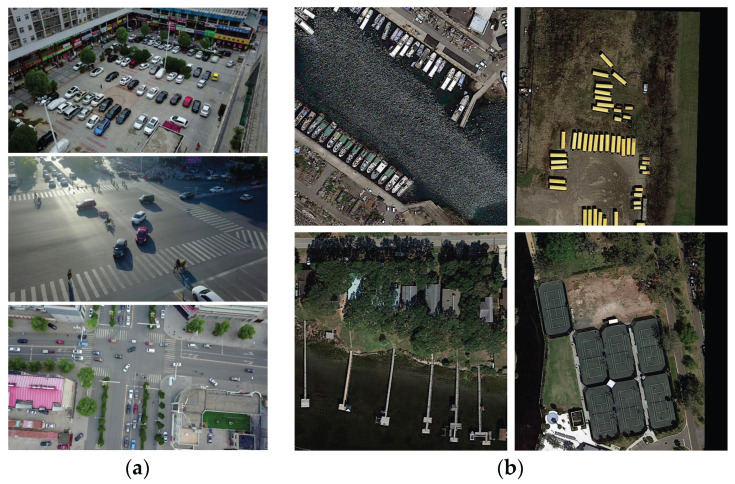
(**a**) Samples images from VisDrone2019 dataset; (**b**) Samples images from DOTAv1.0 dataset.

The VisDrone2019 [43] dataset is a large UAV perspective dataset. In the official data, there are 6471 images in the train set, 1580 images in the test set and 548 images in the validation set, with a total of 11 categories. The samples in this dataset have the following characteristics: (1) the objects in small areas are densely distributed; (2) the background is complex, and there is target occlusion, insufficient light, and interference from objects of a similar shape, etc. (3), and many objects are occluded. Accordingly, this dataset is suitable to verify the performance of the Ghost-YOLOS network in detecting small objects. Some sample pictures of the validation set are shown in Figure 17a.

The DOTAv1.0 [44] dataset contains 2806 aerial images, approximately 4000 × 4000 in size, with a total of 188,282 instances across 15 categories. We split all images into small patches of 1024 × 1024 resolution during training, and used the validation set of this dataset as a test in the experiment. Some sample pictures of the validation set are shown in Figure 17b.

#### 3.1.2. Experimental Environment

The whole experiment is divided into two parts: Ghost-YOLOS lightweight network construction and FPGA hardware acceleration. The network construction and training are mainly completed on a Personal Computer (PC). To be fair, the training parameters of all experiments are set to be the same. The stochastic gradient descent method is used for training, the resolution of input images is adjusted to 416 × 416 through preprocessing, and the batch size is set to 8. All models are trained with 200 epochs. The maximum learning rate is $1\times 10^{-2}$ when the minimum is $1\times 10^{-4}$, and the decreasing mode is cosine decreasing. The experimental environment of PC is detailed in Table 2.

**Figure 18:**
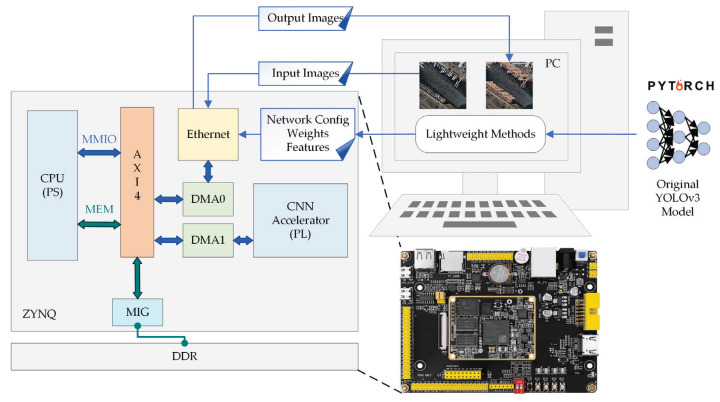
The hardware configuration for the experiment.

To evaluate the performance of the hardware accelerator, the SoC Xilinx ZYNQ Z7020 is used for CNN implementation. In addition to the FPGA chip, the system is equipped with 1 GB DDR3 SDRAM for storing feature maps and weight parameters. The whole project is built based on Vivado 2018.3 and Vivado HLS 2018.3. The Vivado HLS part implemented is based on C++, which is used for high-level synthesis of IP core of the preprocessing and post-processing module. The Vivado part is implemented based on Verilog for the construction of the convolution computing module PE and the synthesis and implementation of the overall project. The clock cycle of the entire system is set at 150 MHz. Figure 18 shows the hardware configuration for the experiment. 

### 3.2. Experimental Results

#### 3.2.1. Ablation Experiment of Ghost-YOLOS Network

**Figure 19:**
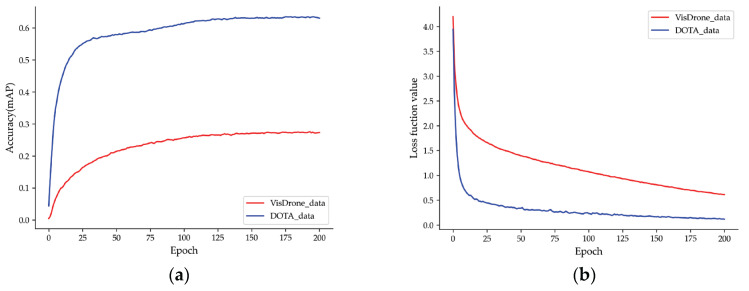
Network training result: (**a**) mAP of the two datasets; (**b**) loss curves of the two datasets.

Ablation experiments are performed to verify the effectiveness of the respective class of improvements in the Ghost-YOLOS network. Figure 19 presents the trend of the mAP and loss function value of DOTA and Visdrone2019 datasets trained using the Ghost-YOLOS network before quantization. All curves are smoothed for better observation of trends.

Table 3 lists the Ablation Experiment results of the two datasets. The original YOLOv3 serves as baseline, and the Ghost-YOLOS is the final version after the improvements are added. Based on the YOLOv3, a total of four improvements are added, expressed in the following.

First, the backbone of YOLOv3 is replaced with GhostNet from DarkNet53. Under the effect of this improvement, the mAP is increased by 7.80%, the number of model parameters is reduced by 61.77%, and the model size is reduced by 72.18% on the DOTA dataset. Additionally, this improvement leads to the increased mAP by 5.41%, the reduced number of model parameters by 61.79%, and the reduced model size by 72.18% on the VisDrone dataset. As indicated by the improvement of a wide variety of indicators, GhostNet outperforms DarkNet53 in YOLOv3, suggesting the effectiveness of this improvement. 

Second, channel pruning is performed on the model to reduce the number of parameters. On the DOTA dataset, this improvement leads to the reduced number of parameters of the model by 71.65% and the reduced size of the model by 85.24%. Moreover, the mAP of the model is reduced by 2.93%. Under the effect of this improvement, the number of parameters of the model is reduced by 72.57% on the VisDrone dataset, and the size of the model declines by 83.18%. Furthermore, the mAP of the model is reduced by 2.86%. Although the overall accuracy is affected, the number of parameters and the size of the model are notably reduced, such that the memory consumption and the difficulty of hardware deployment are reduced.

Third, knowledge distillation is adopted to recover the accuracy lost after model pruning. On the DOTA dataset, this improvement leads to the improved model mAP by 2.42%; on the VisDrone dataset, the model mAP is improved by 2.21%. Compared with the model before pruning, the accuracy loss of the model after knowledge distillation falls into a scope of 0.7%, suggesting the effectiveness of knowledge distillation in accuracy recovery.

Fourth, the 16-fixed quantization of the weights and feature map parameters is performed, thus leading to a reduced model size by approximately 50% on both datasets with a 0.3% loss of accuracy. Through this improvement, the resource consumption of neural network hardware deployment is notably reduced, and the overall inference speed of the network is increased.

#### 3.2.2. FPGA Accelerator Performance

A series of parallelism experiments are set (Table 4) to explore the optimal parallelism degree. To confirm the effectiveness of the proposed depth-wise convolutional computation architecture, a controlled experiment is set without using depth-wise convolutional architectures. 

As revealed by Experiment 1, 2 and 3 with the increase of the parallelism, the throughput of the CNN accelerator is also increased with the usage of the PE units. However, the DSP efficiency reaches its maximum under the ordinary convolution parallelism of 8 × 8. The reason for the above result is that in the Ghost-YOLOS, the output channels of the 16 GBN modules reach 16, 24, 24, 40, 40, 80, 80, 80, 80, 112, 112, 160, 160, 160, 160, and 160. As indicated by this result, the number of output channels is a multiple of 8, such that selecting 8 as the parallel factor can make the convolutional layers with different numbers of channels or step sizes develop a unified data acquisition logic. A total of 11 of the 16 GBN modules have output channels that are multiples of 10, such that choosing a parallel factor of 10 also exhibits high-DSP efficiency, only 0.003 less than a parallel factor of 8. Thus, the proposed CNN accelerator selects 10 as its parallelism factor to achieve higher computational throughput. The comparison of experiments 2 and 4 suggests that the throughput of the CNN accelerator is improved by 33.2% with the depth-wise convolution computing architecture, such that the effectiveness of the design of this study is confirmed.

Table 5 presents the hardware resource consumption of the CNN hardware accelerator. For the full CNN accelerator, the consumption of LUT, FF, BRAM, and DSP reaches 36,528, 30,252, 84, and 113, respectively. Most of the BRAM resources are adopted to cache the feature map and weight parameters in the DDR, and most of the DSP resources are employed to develop the PE computing unit. As indicated by the result of in the ZYNQ7020 with very limited hardware resources, the proposed lightweight network can still be deployed with less than 70% hardware utilization, suggesting that the scheme of this study is promising in the deployment of edge devices.

Moreover, the performance of the CNN accelerator is evaluated for computing power and power consumption. For computing power, Giga Operations per Second (GOPS) serves as the evaluation metric in this study, suggesting the number of operations performed by the accelerator per unit time. Vivado’s internal power estimator is employed for power consumption. The CNN accelerator computing performance reaches 29.53 GOPS at the clock frequency of 150 MHz by inferencing all pictures in the validation set of both datasets. The power consumption of the whole system reaches 2.982 W, where the static power of the hardware is 0.336 W, and the power efficiency is 9.90 GOPS/W. As revealed by the above-described result, the proposed CNN accelerator is capable of meeting a good computational performance overhead with low power consumption, thus conforming to the low-power edge device deployment requirements.

**Figure 20:**
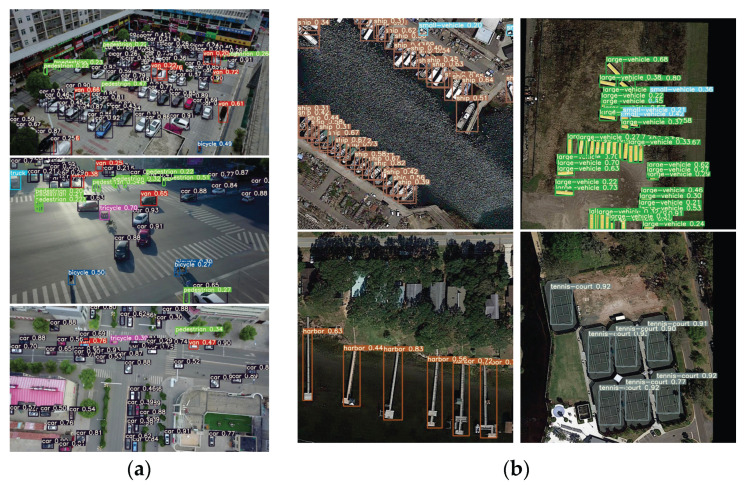
CNN accelerator detection results: (**a**) from Visdrone2019 dataset, and (**b**) from DOTAv1.0 dataset.

In addition, the target detection performance of the accelerator is examined (e.g., inference time and detection accuracy). For the DOTA dataset, 5297 images in the validation set are tested, and an average inference time of 0.32 s per image, a mAP of 62.58%, and a total accuracy loss of less than 0.5% are finally obtained compared with the original model. For the VisDrone2019 dataset, 548 images in the validation set are tested, and an average inference time of 0.28 s per image, a mAP of 26.55%, and a total accuracy loss of less than 0.6% are obtained compared with the original model. The CNN accelerator is capable of completing the network inference with a small loss of accuracy, and the fast speed can also conform to the real-time requirements. Figure 20 illustrates some detection results.

Then, we compared the proposed CNN accelerator computing power with that the CPU and GPU, as shown in Table 6. The power consumption of the CPU and GPU in network inference is 95 W and 250 W, respectively, which are 32× and 84× higher than the power consumption of the 2.98 W proposed CNN accelerator. Compared with a high-power CPU and GPU, our FPGA is more suitable for low-power edge device deployment.

Although the throughput of CPU and GPU is higher than that of the proposed CNN accelerator, both of them perform worse than the proposed CNN accelerator for power efficiency. It can be seen that, with a batch size equal to 1, although the CPU and GPU clock frequencies are 24.7× and 9.0× higher than that of FPGA, respectively, the proposed CNN accelerator achieves 29.11× better power efficiency than that of CPU, and even achieves about 5.29× better power efficiency than that of GPU. The high power efficiency represents the excellent computational performance of the proposed CNN accelerator, making it not only capable of being deployed in edge devices with limited resources, but also capable of completing faster computations with less power.

For object detection tasks, the accuracy of the proposed CNN accelerator falls into a scope of 0.1% compared with that of the CPU and GPU. We consider that such accuracy loss is mainly due to the hardware optimization of the SE module and the accuracy limitation of floating-point arithmetic, and this accuracy loss is acceptable in practical applications.

### 3.3. Performance Comparison

To better show the performance of the proposed lightweight network and hardware accelerator, we set up a series of comparative experiments. First, we compared the performance of the Ghost-YOLOS network with other networks on the same data set, including accuracy, calculated amount, and number of parameters, as shown in Table 7. We compared the performance of the Ghost-YOLOS network with some classical models [45,46,47,48], and some recent innovative models [49,50,51,52,53] on the DOTA and VisDrone datasets. 

For the DOTA dataset, The RetinaNet used in Ref. [45] has considerable parameters and its accuracy is not as good as some commonly used networks. The YOLOv4-tiny network used by Ref. [46] has 0.8 M fewer parameters than our Ghost-YOLOS, but its accuracy is 4.4% lower than our network. The YOLOv5s network used by Ref. [47] has 0.7% higher accuracy than Ghost-YOLOS. However, the number of parameters and GFLOPs are 0.5 M and 2.17 higher than those of Ghost-YOLOS, respectively, and the YOLOv5s network has considerable residual structures, which will increase the number of memory accesses in hardware, and is not conducive to the acceleration of network inference. Refs. [45,49] have improved on the original network and achieved better detection performance. However, the improvement of Faster-RCNN in Ref. [45] still has the characteristic that the number of parameters is too large. The R2-YOLOS proposed in Ref. [49] performs better than our network for detection accuracy, but its parameter quantity and computation amount are 1.67× and 3.40× more than that of Ghost-YOLOS, respectively, which is too large to meet the real-time detection requirements of edge equipment. Compared with the conventional two-stage detection algorithm, the one-stage detection algorithm adopted by Ghost-YOLOS has a smaller size and less computation, and because of the replacement of the backbone, the model can achieve higher accuracy.

For the VisDrone dataset, we obtained the best performance from the Refs. [45,48,51,52,53]. We achieved the highest accuracy of 26.6% with the lowest parameter count of 5.8 M and GFLOPs of 7.63. This is because the splicing structure of the Ghost module and the SE attention mechanism make our network have a stronger effect on small target detection. The performance of our network in two datasets shows that Ghost-YOLOS can play a good performance in aerial and UAV object detection, and its lightweight calculation and parameter amount makes it very suitable for deployment in edge devices with limited computing resources.

In addition, we compared the proposed CNN accelerator with previous works [54,55,56,57,58,59], as shown in Table 8. Ref. [54] proposed a CNN accelerator which used network compression and layer-chaining techniques. Ref. [55] designed a highly flexible and reconfigurable accelerator, which mainly focuses on the problem that transposed convolution, dilated convolution and depth-wise convolution have low utilization in the common convolution engine. Ref. [56] proposed an energy-efficient architecture for super-resolution images. However, the hardware implementation in Refs. [54,55,56] consumes 1536, 607, and 1512 DSPs, respectively, which is 13.6×, 5.4×, and 13.4× more than that of the proposed solution. It is difficult to implement in the case of limited hardware resources, and their DSP efficiency is lower than the proposed CNN accelerator. Ref. [57] designed a composite hardware accelerator, which proposed a multiple single computing engine architecture based on a row-level pipelined streaming strategy. The accelerator achieves the TOPS-level of throughput with extremely high DSP efficiency. However, such excellent performance brings great hardware resource consumption. The design consumes 3395 DSPS and reaches 14.36 W power, which is 30.04× more and 4.8× higher than that of our accelerator, respectively. This limits its application in power-sensitive scenarios. Ref. [58] proposed a self-adaptive CNN accelerator, which can modify its architecture while inferencing the networks. Ref. [59] proposed an automatic CNN deployment solution on resource-limited devices. Both works [58,59] used less DSP resources to implement the inference of the CNN. However, the accelerator in Refs. [58,59] only achieves 10.25 GOPS and 22.17 GOPS throughput, respectively. Compared with the design where the whole network is repeatedly stacked with modules in the order of the respective layer, the proposed CNN accelerator can perform different types of convolution computing using a single PE array, such that the efficiency of hardware resources can be significantly increased.

In brief, our accelerator has lower power consumption and higher DSP efficiency in the identical scale processing engine. Compared with the previous works, the proposed CNN accelerator has a better balance in throughput, resource and power, and is more suitable for deployment in edge devices with limited resources and strict power requirements.

## 4. Discussion

The YOLO series network has evolved into numerous versions thus far, and YOLOv3 does not exhibit the optimal performance among these versions. However, it is selected as the original network in this study due to the comprehensive consideration of the throughput and resources of the accelerator. The network backbone after YOLOv2 has an excessive amount of residual blocks, which will hinder hardware processing. Cross-layer operation represents multiple memory access for hardware, which will cause extra memory overhead and hardware resource consumption (especially LUT and FF). Accordingly, its backbone network is replaced with GhostNet. Subsequently, special attention should be paid to the neck and head parts of YOLO. YOLOv4 employs SPP (Spatial Pyramid Pooling) + PANet (Path Aggregation Network) for the neck part compared with YOLOv3, which improves the precision while increasing the network depth and the number of parameters. YOLOv5 adds more residual structure and concatenate modules, thus hindering the hardware implementation of the network.

Moreover, GhostNet exhibits excellent performance on YOLOv3 by significantly reducing its parameter count while achieving comparable accuracy to YOLOv5s. However, the performance of YOLOv4 and YOLOv5 is not significantly improved. Experiments are performed on the DOTA dataset, and the results suggest that YOLOv4-Ghost only achieves a 1.6% increase in accuracy compared with the original network, while YOLOv5-Ghost only achieves an improvement of 0.7%. Given hardware resource consumption and performance comprehensively, the YOLOv3 network is ultimately selected as the base model. This decision is made following a thorough evaluation of multiple factors, which comprises the trade-off between hardware feasibility, resource constraints, accuracy, and performance. Although the subsequent versions of the YOLO network may exhibit increased accuracy, our choice of YOLOv3 as the original network best conforms to specific requirements.

After the original network is lightened, the Ghost-YOLOS network is deployed into Xilinx ZYNQ Z7020, and a series of hardware optimizations are performed for network characteristics. 1. Because depth-wise convolution and common convolution exist in Ghost-YOLOS network, a reconfigurable convolution operation array is designed to save hardware resources. 2. To reduce the delay of data transmission between memory and processing unit, a series of memory access optimization schemes are designed for BRAM. 3. Due to the poor hardware friendliness of SE attention mechanism, the approximate implementation of activation function and optimization scheme of maximum pooling. As indicated by the experimental results, our optimization scheme can improve the throughput and energy efficiency of the accelerator.

After comparison, certain limitations are identified in the application of proposed lightweight network and CNN accelerator:

- The pruning and distillation methods used in this study are strictly affected by the model and cannot improve the detection accuracy of the model itself, so it is necessary to use more lightweight methods targeted at the model structure.
- Floating-point computation is not applicable to FPGA, so data quantization is very necessary. However, the quantization method employed in this study inevitably causes precision loss, such that a more fine-grained quantization method should be developed to reduce the effect on precision.
- The solution in this study proposes a reconfigurable convolution computing array to perform convolution computing of different modes. However, the hardware resource limitation of the FPGA applied leads to the inability of parallelism to expand to a higher dimension, such that inevitable resource redundancy is caused. Accordingly, chips with richer hardware resources may achieve higher resource utilization efficiency.

Based on the innovation and limitation of the proposed solution, several possible uses and effects are indicated:

- The proposed accelerator operators include common convolution, deep convolution, SE attention mechanism, full connection and Max pooling, etc., which are not only suitable for Ghost-YOLOS network, but also applicable to any network consisting of stacks of these modules (e.g., MobileNet and ShuffleNet). Hardware acceleration of this type of network can be achieved only by changing the task scheduling at the PS side, which offers novel ideas for the application of FPGA technology in deep learning.
- The proposed solution has the characteristics of low power consumption, high energy efficiency, high throughput and high precision, such that it can be applied to the field of remote sensing object detection. Additionally, it can be integrated with advanced sensors, Internet of Things (IoT) and human–computer interaction technologies (e.g., mobile robots and exploration vehicles) to expand its application prospects.

## 5. Conclusions

In this study, a lightweight remote sensing image detection model, Ghost-YOLOS, and its energy-efficient CNN accelerator solution are proposed based on the FPGA, which comprises model optimization methods and a hardware acceleration architecture.

Through the optimization of the model, the model parameters and size are primarily reduced as much as possible, while the accuracy can be ensured, with the aim of adapting to the edge devices deployment. Accordingly, GhostNet, a lightweight feature extraction network, is introduced, and channel pruning is conducted on the whole model. A knowledge distillation method is adopted to recover the accuracy lost by the parameter number reduction. Lastly, 16-bit fixed-point quantization is performed prior to deployment in the hardware.

An efficient CNN accelerator is proposed for hardware acceleration. A reconfigurable PE array with high parallelism is developed in accordance with the characteristics of different convolution in the Ghost-YOLOS network, which is capable of performing different types of convolution computing according to the control signal. The SE attention mechanism in the GBN module is optimized at the hardware level, comprising 2D pooling separation and Sigmoid activation function hardware implementation. For data storage optimization, different storage methods are designed following to the characteristics of feature maps and weights, and a dual cache design is implemented to save memory access overhead.

Compared with the design that the whole network is repeatedly stacked with modules in the order of the respective layer, the CNN accelerator developed in this study exhibits the structure of a single convolution computing array, which is capable of performing different types of convolution computing based on the control signal, such that the on-chip physical resources can be saved, with the aim of carrying out more dimensions of parallelism expansion.

In general, in comparison with the original YOLOv3 network, the mAP of the Ghost-YOLOS on the aerial dataset DOTAv1.0 is increased by 7.04%, the number of parameters only accounts for 10.84% of the original model, and the model size is reduced to 5.08%. Moreover, on the UAV dataset VisDrone2019, the mAP of the Ghost-YOLOS is improved by 4.54%, the number of parameters only takes up 9.45% of the original model, and the model size declines to 6.21%. Accordingly, the Ghost-YOLOS outperforms YOLOv3 in efficiency, and it more significantly applies to deployment on edge devices with limited computing resources.

Subsequently, the Ghost-YOLOS network is well deployed on Xilinx ZYNQ Z7020 using the proposed CNN acceleration scheme. As indicated by the experimental results, the power of the proposed CNN accelerator only reaches 2.98 W, and the DSP consumption only reaches 113. The object detection of the two datasets achieves the throughput of the CNN accelerator of 29.53 GOPS, the power efficiency of 9.90 GOPS/W, as well as the DSP efficiency of 0.261 GOPS/DSP. As indicated by the comparison with the previous works, the proposed solution is characterized by low power consumption, low resource consumption, and high efficiency, thus becoming promising in the deployment of remote sensing edge devices with strict power requirements and limited resources.

In further research, the existing pruning and knowledge distillation schemes will be replaced, and more lightweight methods will be designed for the network structure. Moreover, the hardware of higher versions of YOLO networks will be optimized, and more hardware-friendly quantization schemes will be designed.

## Figures and Tables

**Table 1:** The fitting function after piecewise quadratic approximation for FPGA.

Interval	Fitting Function	Fitting Function for FPGA
(0, 0.5]	f(x)=0.2452x+0.5005	f(x)=(1004x+2050)/4096
(0.5, 2.45]	$f(x)=-0.0446x^{2}+0.2852x+0.4908$	$f(x)=(-183x^{2}+1168x+2010)/4096$
(2.45, 4]	$f(x)=-0.0172x^{2}+0.1506x+0.6533$	$f(x)=(-70x^{2}+617x+2676)/4096$
(4, 5.6]	$f(x)=-0.0039x^{2}+0.0483x+0.8503$	$f(x)=(-16x^{2}+198x+3483)/4096$
(5.6, 7.5]	f(x)=0.0018x+0.9862	f(x)=(7x+4039)/4096
(7.5, +∞)	f(x)=1	f(x)=4096/4096

**Table 2:** Experimental environment of PC.

Environment	Versions or Procedure
CPU	Intel(R) Core(TM) i7-8700 K, 3.70 GHz
GPU	NVIDIA GeForce RTX 2080 Ti, 11 GB Memory
CUDA	v11.3
Python	v3.9.7
PyTorch	v1.11
OS	Windows 10

**Table 3:** Ablation experiment results on the two datasets.

Model	Dataset	mAP (%) (*,*)	Parameters (M) (*)	Size (MB) (*)
YOLOv3	DOTA v1.0	55.61	61.52	248.0
+GhostNet		63.41 (+7.80, +7.80)	23.52 (38.23%)	179.0 (72.18%)
+Model Pruning		60.48 (−2.93, +4.87)	6.71 (10.91%)	25.3 (10.20%)
+Knowledge Distillation		62.90 (+2.42, +7.29)	6.67 (10.84%)	25.1 (10.12%)
+Quantization		62.65 (−0.25, +7.04)	6.67 (10.84%)	12.6 (5.08%)
YOLOv3	VisDrone2019	22.07	61.48	248.0
+GhostNet		27.48 (+5.41, +5.41)	23.49 (38.21%)	179.0 (72.18%)
+Model Pruning		24.62 (−2.86, +2.55)	5.85 (9.52%)	30.2 (12.18%)
+Knowledge Distillation		26.83 (+2.21, +4.76)	5.81 (9.45%)	30.1 (12.13%)
+Quantization		26.61 (−0.22, +4.54)	5.81 (9.45%)	15.4 (6.21%)

Note: (*,*) represents (mAP increased by this model compared with the previous model, mAP increased by this model compared with the initial model); (*) represents (the percentage of the model parameters compared with the initial model).

**Table 4:** The performance of CNN accelerator with different parallelism degrees.

Experiment	1	2	3	4
Common Convolution Parallelism (C × R)	8 × 8	10 × 10	13 × 13	10 × 10
Depthwise Convolution Parallelism (P × R × Q)	9 × 2 × 3	9 × 2 × 6	9 × 6 × 3	N/A
Resource Redundancy	7	8	7	N/A
Throughput (GOPS)	18.24	29.53	36.48	22.16
DSP Blocks	69	113	174	105
DSP Efficiency (GOPS/DSP)	0.264	0.261	0.210	0.211

Note: Resource Redundancy means the difference between Common Convolution Parallelism and Depthwise Convolution Parallelism, which represents the amount of redundant resources in one operation when the hardware resources of the other operation are fully utilized.

**Table 5:** The hardware resource consumption of the CNN hardware accelerator.

Resource	LUT	FF	BRAM_36K	DSP
Available	53,200	106,400	140	220
Consumption	36,528	30,252	84	113
Utilization (%)	68.66	28.43	60.00	57.73

**Table 6:** Evaluation results on CPU, GPU and proposed CNN accelerator.

	CPU	GPU	FPGA
Device	Intel(R) Core (TM) i7-8700 K	NVIDIA GeForce RTX 2080 Ti	Xilinx ZYNQ Z7020
Technology (nm)	14	10	28
Frequency (MHz)	3700	1350	150
Power (W)	95	250	2.98
Batch Size	1	1	1
mAP (%) ^1^	62.65	62.65	62.58
Throughput (GOPS)	32.35	467.22	29.53
Power Efficiency (GOPS/W)	0.34	1.87	9.90
Power Efficiency Ratio	1×	5.50×	29.11×

Note: ^1^ The mAP is for DOTA dataset to evaluate the performance of the three devices. Batch Size means the number of samples used for an inference.

**Table 7:** Comparison of performance on the DOTA and VisDrone dataset.

Model	Backbone	Dataset	mAP (%)	Parameters (M)	GFLOPs
IMP-FRCNN [49]	ResNet50	DOTA v1.0	58.0	N/A	134.5
RetinaNet [45]	ResNet101		38.9	55.4	179.5
YOLOv4-tiny [46]	CSPDarknet53		58.3	5.9	N/A
YOLOv5s [47]	CSPRepResNet		63.5	7.2	10.58
R2-YOLOS [50]	ResNet50		66.3	11.2	28.58
Ghost-YOLOS (ours)	GhostNet		62.7	6.7	8.41
FRCNN [48]	ResNet50	VisDrone 2019	13.5	41.17	134.5
RetinaNet [45]	ResNet101		8.9	55.4	179.5
RFNet [51]	ResNet50		15.2	79.57	N/A
VAMYOLOX-X [52]	CSPDarkNet53		24.5	104.6	510.4
LV-YOLOv5 [53]	CSPRepResNet		25.6	36.6	38.8
Ghost-YOLOS (ours)	GhostNet		26.6	5.8	7.63

Note: N/A means we did not find the exact data in the references.

**Table 8:** Comparison of proposed CNN accelerator with previous works.

Architecture	[54]	[55]	[56]	[57]	[58]	[59]	Our work
FPGA	Intel Arria 10	Intel Arria 10	Xilinx XC7K410T	VX980T	Xilinx ZYNQ Z7020	Xilinx AC701	Xilinx ZYNQ Z7020
Model	StyleNet	ENet	CNN+ DCNN	VGG16	Tiny YOLO	IMP YOLOv2	Ghost-YOLOS
Network Complexity (GFLOPS)	37.60	3.68	3.30	15.47	5.41	379.55	8.41
Quantization	16-Fixed	16-Fixed	13-Fixed	8/16-Fixed	16-Fixed	8-Fixed	16-Fixed
Frequency (MHz)	200	200	130	150	150	200	150
DSP Blocks	1536	607	1512	3395	99	94	113
Throughput (GOPS)	335.0	97.2	390	1000	10.25	22.17	29.53
Power (W)	5.90	N/A	5.38	14.36	N/A	3.407	2.98
Power Efficiency (GOPS/W)	56.76	N/A	72.49	69.64	N/A	6.51	9.90
DSP Efficiency (GOPS/DSP)	0.218	0.160	0.258	0.295	0.104	0.236	0.261

Note: N/A means we did not find the exact data in the references.

## Data Availability

Not applicable.

## Abbreviations

The following abbreviations are used in this manuscript:

CNN	Convolutional Neural Network
FPGA	Field Programmable Gate Array
GPU	Graphics Processing Unit
CPU	Central Processing Unit
SE	Squeeze and Excitation
BN	Batch Normalization
ASIC	Application Specific Integrated Circuit
MACC	Multiplication Accumulators
PE	Processing Engine
WPE	Window Processing Engine
BPE	Block Processing Engine
DDR	Double Data Rate
SDRAM	Synchronous Dynamic Random-Access Memory
BRAM	Block Random-Access Memory
PL	Programmable Logic
PS	Processing System
mAP	mean Average Precision
GOPS	Giga Operations per Second
DSP	Digital Signal Processor
LUT	Look-Up Table
FF	Flip-Flop
PC	Personal Computer
R&D	Research and Development
IoT	Internet of Things
